# Plant Antimicrobial Peptides and Their Main Families and Roles: A Review of the Literature

**DOI:** 10.3390/cimb47010001

**Published:** 2024-12-24

**Authors:** Samuel Salomão Silva de Oliveira, Milena Bellei Cherene, Gabriel Bonan Taveira, Érica de Oliveira Mello, André de Oliveira Carvalho, Valdirene Moreira Gomes

**Affiliations:** Laboratório de Fisiologia e Bioquímica de Microrganismos, Universidade Estadual do Norte Fluminense Darcy Ribeiro, Rio de Janeiro 28013-602, Brazil; samuelsalomao01@gmail.com (S.S.S.d.O.); bioblogmilena@gmail.com (M.B.C.); gabrielbtaveira@yahoo.com.br (G.B.T.); erica@uenf.br (É.d.O.M.); andre@uenf.br (A.d.O.C.)

**Keywords:** plant defense, bioactive peptides, plant antimicrobial peptides

## Abstract

Antimicrobial peptides (AMPs) are constituent molecules of the innate defense system and are naturally produced by all organisms. AMPs are characterized by a relatively low molecular weight (less than 10 kDa) and a variable number of cysteine residues that form disulfide bonds and contribute to the stabilization of the tertiary structure. In addition, there is a wide repertoire of antimicrobial agents against bacteria, viruses, fungi, and protozoa that can provide a large number of prototype peptides for study and biochemical manipulation. In this sense, plant AMPs stand out because they have a wide range of biological functions against microorganisms and potential applications in medicine and agriculture. Herein, we describe a mini-review of the principal AMP families, such as defensins, lipid transfer proteins (LTPs), thionins, heveins, and cyclotides. The objective of this work was to present the main discoveries regarding the biological activities of these plant AMP families, especially in the last 20 years. We also discuss the current knowledge of their biological activities, gene expression, and possible uses as antimicrobial molecules and in plant biotechnology.

## 1. Introduction

### 1.1. Antimicrobial Peptides: Brief History and Mechanism of Action

Antimicrobial peptides (AMPs) play a role in the defense and are naturally produced by all organisms, from bacteria to plants, vertebrates, invertebrates, and even eukaryotic cell populations [1,2]. AMPs are characterized by their small size (10 to 100 amino acid residues), compact structure, rigidity, and resistance to denaturation by temperature and pH. In addition, they have a broad repertoire of antimicrobial activities against bacteria, viruses, fungi, and protozoa and can provide a large number of prototype peptides for study and biochemical manipulation [3].

In 1939, an antimicrobial peptide was discovered in prokaryotic cells that lyse the living cells of a wide range of Gram-positive bacteria, providing protection for mice against pneumococcal infection. This peptide was later called gramicidin and became the first antimicrobial peptide; it is a nonribosomal peptide that was clinically tested and commercially manufactured as an antibiotic agent [1,2].

In 1941, tyrocidin was discovered and shown to have antimicrobial effects on other forms of Gram-positive bacteria in general. In 1942, an antimicrobial peptide was isolated from wheat triploid endosperm tissue, later called purothionin, and was considered effective against *Pseudomonas solanacearum*, *Xanthomonas campestris*, and *Corynebacterium michiganense* [4,5].

In plants, several other peptides with ribosomal synthesis were isolated and characterized almost 40 years after the isolation of purothionin from wheat, including cyclotides, snakins, lipid transfer proteins, hevein, puroindolines, and defensins, among others [6,7].

AMPs provide the first line of defense against invading pathogens and are constitutively produced and/or induced when an active infection or endotoxin is detected by host cells. They are cationic molecules due to their high content of lysine and arginine and are organized in an amphipathic structure. They have hydrophilic and hydrophobic ends, providing solubility in both aqueous and hydrophobic environments. AMPs electrostatically bind to specific targets present on the cell membrane that have a negative surface charge. AMPs have a positive charge that varies between them, and this net charge is highly important for determining the affinity of a molecule for the anionic site of the target [1,8].

AMPs contain hydrophobic residues that are inserted into the lipid bilayers of the cell membrane to mediate permeabilization and membrane rupture, leading to rapid cell death [6,7]. Cell membrane permeabilization is the most common mechanism of action; however, some AMPs, such as buforin II, spontaneously pass through the membrane, inhibiting cell functions by binding to cell DNA and RNA [9,10,11].

Some AMP molecules demonstrate no secondary structure in an aqueous environment but assume a secondary structure when exposed to lipids, such as those present in bacterial cell membranes [1]. Electrostatic attraction between AMPs and membrane lipids occurs through teichoic and lipoteichoic acids in Gram-positive bacteria or through lipopolysaccharides in Gram-negative bacteria. This attraction results in instability of the outer membrane, which allows the translocation of AMPs across the membrane, causing bacterial lysis. There are different models by which AMPs can damage pathogen membranes, such as the barrel model, toroidal pore model, and carpet model [12].

Studies on the interactions between AMPs and bacterial membranes demonstrate that it is difficult for bacteria to develop resistance to AMPs, supporting the wide application of AMPs in livestock, food production, and drugs to avoid the excessive use of antibiotics [13]. Because microbial resistance to conventional drugs involves drastic changes in membrane phospholipid composition, affecting multiple systems in turn, AMPs do not contribute to the emergence of resistant pathogens [14]. The diverse mechanisms of action of AMPs and the selectivity of antimicrobial agents for microbial cells give AMPs a broad spectrum of effects against Gram-negative and Gram-positive bacteria, fungi, parasites, viruses, and tumor cells. In addition, some AMPs mediate chemotaxis, apoptosis, immunomodulatory effects, and wound healing, which make them suitable for the development of new therapeutics [14,15].

Natural cationic amphipathic peptides have potential in the development of multiple drugs. They exhibit low toxicity and/or hemolytic activity toward mammalian cells; however, as concentrations increase, they may become unsuitable for direct use in therapeutic development, because their cytotoxic effect will be greater than their therapeutic effect [13].

Hydrophobicity is related to the cytotoxicity of AMPs because the more hydrophobic the peptide is, the greater its ability to penetrate cell membranes, affecting prokaryotic and eukaryotic cells. Toxicity can occur by binding to cell membranes, leading to hemolysis or cytotoxicity. The cytotoxicity of the peptide is likely associated with its hydrophobicity, amphipacity, and helicity [16]. Therefore, certain chemical modifications are needed to enable the development of AMPs as therapeutic antimicrobial agents [16].

This review was designed in response to the growing body of research on plant AMPs over the past two decades, with the aim of organizing and synthesizing key findings related to the major families of plant AMPs and their biological activities. To achieve this goal, we conducted a comprehensive literature search using the databases Google Scholar, the Antimicrobial Peptide Database (APD), and PubMed, covering the period from 2002 to 2023. Keywords such as “plant antimicrobial peptides”, “peppers”, “Capsicum”, “thionin”, “hevein-like”, “defensins”, “lipid transfer proteins”, and “cyclotides” were employed to identify relevant academic papers. Studies were included if these terms appeared in titles, abstracts, or keywords, with a focus on articles that explored the biological activities of AMPs. The novelty of this review lies in its structured, chronological presentation of the evolution of plant AMP research, particularly emphasizing its biotechnological potential. This work offers a more detailed timeline and analysis of AMP families, providing a comprehensive resource for understanding key trends and advancements in this field. Moreover, these findings highlight gaps in the current knowledge, suggesting targeted approaches for future research on the biological functions of plant AMPs.

### 1.2. Classification and Structure of AMPs

**Figure 1:**
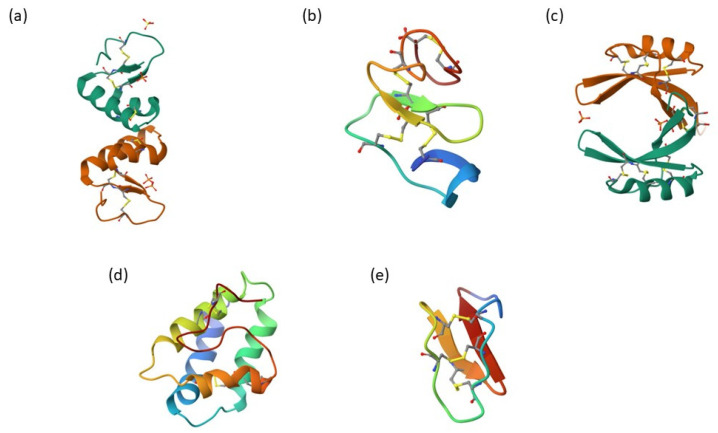
(**a**) Thionin from *Viscum album* L. (PDB: 1OKH). (**b**) Hevein-like from *Gypsophila vaccaria* (PDB: 5XDI). (**c**) Defensin from *Oryza sativa* (PDB: 6LCQ). (**d**) LTP from *Hordeum vulgare* (PDB: 1LIP) (**e**) Cyclotides from *Oldenlandia affinis* (PDB: 2M9O).

AMPs can be classified into four families based on their three-dimensional structure according to the Antimicrobial Peptide Database (ADP): alpha (α), beta (β), alpha-beta (αβ), and non-alpha-beta (nonαβ) (Figure 1). The alpha family is composed of AMPs with helical structures (e.g., magainins and LL-37). Beta family peptides are composed of AMPs with at least one β-sheet in their structure (e.g., human α-defensins and lactoferricidin). AMPs from the alpha-beta family have both α-helices and β-sheets in their 3D structure (e.g., β-defensins), while AMPs from the non-alpha-beta family contain neither α- α-helices nor β-sheets in their structure; for example, indolicidin [17].

Furthermore, some authors divide AMPs based on their secondary structure into α-helixes, β-sheets, and extended peptides. α-helices AMPs have a linear structure when in an aqueous solution but become an amphipathic helical structure when in contact with the bacterial membrane or organic solvents because they contain hydrophobic residues capable of interacting with various types of membranes. The α-helical structure leads to high affinity between peptides and microbial membranes, resulting in better permeabilization of bacterial membranes [18,19,20,21]. This group of AMPs is mostly less than 40 amino acids in length; for example, cecropins (from the *Hyalophora cecropia* L. insect), magainin (from the clawed frog *Xenopus laevis* Daudin), and pleurocidin (isolated from several fish) [22,23,24].

β-sheet AMPs are generally cyclic molecules formed by at least two antiparallel β-sheets stabilized by disulfide bonds between conserved cysteine residues, forming a rigid structure, as in the defensin family [18,19,20,21,25]. Extended peptides do not have regular secondary structures, as they are stabilized only by hydrogen bonds and van der Waals interactions that induce membrane permeabilization. These peptides are often rich in specific amino acids, such as glycine, arginine, tryptophan, proline, and histidine residues [26,27,28].

AMPs are also classified according to their biological activities as antibacterial, antibiofilm, anti-endotoxin, antitoxin, antiviral, anti-HIV, antifungal, antiparasitic, antimalarial, anticancer, antidiabetic, anti-inflammatory, insecticidal, protease inhibitor, antioxidant, or synergistic. In addition, these methods can be based on the biological source of bacterial AMPs, plant AMPs, and animal AMPs, among others [29,30].

This work aims to provide a comprehensive overview of the different families of AMPs, including their biological activities and potential applications in medicine and agriculture. This review article identifies the principal groups of plant AMPs that have been the subject of the most extensive research over the past two decades and contributes as a reference source for future studies involving biotechnological applications of plant antimicrobial peptides.

## 2. Plant Antimicrobial Peptides

Plant AMPs have high genetic variability and thus greater diversity and ability to recognize different targets [26,27,28]. Most plant AMPs have positive charges that are fundamental for interaction with pathogen membrane lipids [31]. The composition of plant AMPs confers protection against invading pathogens through membrane rupture and pore formation, which promote interaction and interference with fundamental proteins in microbial processes [6,32].

In addition, peptide-based plant genetic engineering holds significant promise for enhancing crop productivity through enhanced resistance against pests and pathogens. Numerous prior studies have demonstrated the remarkable efficacy of transgenic plants that overexpress AMP genes, resulting in heightened resistance to pathogen-induced assaults. 

Plant AMPs are commonly grouped into different families. The most studied of these in the last 20 years have been thionins, defensins, hevein-like proteins, lipid-transfer proteins (LTPs), and cyclotides [33,34].

Table 1 is a compilation of representative peptides from each family of plant AMPs, as well as their name, origin, biological activity, and target.

### 2.1. Thionins

Thionins, which are short plant peptides (~5 kDa) consisting of 45 to 48 amino acid residues, have been identified in a variety of plant species, including cereals, mistletoe, mustard, and poppy [33,34]. Thionins, a term encompassing α/β-thionins and γ-thionins, denote two clearly delineated groups of plant peptides. Despite their shared nomenclature and probable distant ancestral origin, these peptide classes possess dissimilar three-dimensional structures. The three-dimensional structures of thionins, which are small, cysteine-rich antimicrobial peptides. Structurally, thionins are amphipathic molecules. The long arm of this structure consists of two antiparallel α-helices, while the short arm comprises two parallel β-strands. This arrangement forms a groove between the two domains, which is thought to be crucial for their antimicrobial activity (Figure 1a) [7]. Hence, recognizing these proteins as distinct protein families is appropriate, as γ-thionins are more accurately classified as plant defensins. This distinction emphasizes the significance of comprehending the structural attributes and exceptional antimicrobial properties of these materials [6,7,117].

The initial discovery of a plant, thionin, occurred in 1942 when it was isolated from wheat flour and is commonly referred to as purothionin [118]. Thionins quickly gained recognition for their potent toxicity against a range of organisms, including bacteria, fungi, insect larvae, and both animal and plant cells [7]. These hydrophobic peptides engage directly with hydrophobic residues and membrane protein receptors, and their three-dimensional structure is closely tied to their antimicrobial activity. Notably, thionins interact with negatively charged phospholipids present in the cell membrane, disrupting its fluidity and ultimately leading to cell membrane lysis [119,120]. This intricate relationship between structure and function sheds light on the remarkable antimicrobial potential of thionins.

Peptides from the thionin family sourced from black cumin seeds (*Nigella sativa* L.) of the Ranunculaceae family were successfully isolated and characterized. These peptides demonstrated remarkable inhibitory effects against filamentous fungi, specifically *Aspergillus ochraceus* and *Aspergillus fumigatus*, as evidenced by in vitro assays. Additionally, the cytotoxic effects of these compounds were assessed in both tumor and normal cell lines, the results of which were promising and consistent with previous studies that reported nearly 90% cell death induction in RD and Jukart cell lines [39,50]. In another investigation, scientists isolated a genomic clone of a thionin from opium poppy (*Papaver somniferum* L.) of the Papaveraceae family. The expression of this thionin in *Escherichia coli* revealed its noteworthy in vitro antimicrobial activity against *Fusarium oxysporum* and *Botrytis cinerea* fungi [120].

**Figure 2:**
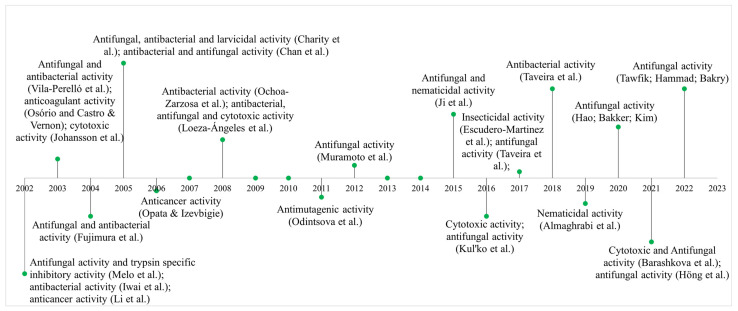
Timeline of scientific papers about main biological activities and gene expression of thionin-type AMPs from 2002 to 2023 [35,36,37,38,39,40,42,43,44,45,46,47,48,49,50,51,52,117,120,121,122,123].

The thionin family has been the subject of extensive research in the past two decades, leading to the elucidation of numerous biological activities and gene expression in transgenic plants (Figure 2). Notably, a thionin derived from cowpea (*Vigna unguiculata* L.) of the Fabaceae family has been found to possess selective inhibitory activity against trypsin, as demonstrated in studies conducted by Melo et al. in 2002 [40].

Thionin isolated from walnuts (*Pyrularia pubera* M.) belonging to the Santalaceae family exhibits a broad spectrum of antimicrobial activities. Specifically, the thionin *P. pubera* thionin (Pp-TH) has been demonstrated to have in vitro activity against various bacteria, including Gram-negative strains such as *Rhizobium meliloti*, *X. campestris* pv. *translucens*, and *X. campestris* pv. *campestris*, as well as Gram-positive bacteria such as *C. michiganensis*. Furthermore, Pp-TH also exhibited antifungal activity against phytopathogenic fungi, including *Pseudonebularia cucumerina*, *F. oxysporum*, and *B. cinerea* [44]. Additionally, within Pp-TH, a segment known as the serine nonapeptide (SNP) has been identified. This SNP segment has been found to exert an effect on the prothrombinase assay, stimulating the activity of the prothrombinase complex, which plays a crucial role in blood clotting [45]. In other studies, researchers discovered and extracted peptides named Tu-AMP 1 and Tu-AMP 2 from tulip bulbs (*Tulipa gesneriana* L.) belonging to the Liliaceae family. These peptides, which are part of the thionin family, exhibited potent antimicrobial effects on a range of bacteria (*Pectobacterium carotovora*, *Agrobacterium radiobacter*, *Agrobacterium rhizogenes*, *Clavibacter michiganensis*, and *Curtobacterium flaccumfaciens*) as well as pathogenic fungi (*F. oxysporum* and *Geotrichum candidum*) [51].

In addition to their antifungal, antibacterial, larvicidal, insecticidal, and nematicidal activities, these antimicrobial peptides exhibit various other biological properties. Within the thionin family, ligatoxin B, isolated from the mistletoe *Phoradendron liga* (Gill.) Eichl. of the Viscaceae family, commonly known as European mistletoe, shares a three-dimensional structure with viscotoxins and purothionins. These peptides have demonstrated cytotoxic effects on the human lymphoma cell line U-937-GTB and the primary multidrug-resistant renal adenocarcinoma cell line ACHN in vitro. Moreover, they are known to be a group of DNA-binding proteins [46].

Another group of proteins, called foratoxins, which belong to the thionin family, was isolated from the leafy mistletoe or Christmas mistletoe (*Phoradendron tomentosum* (DC.) Engelm. ex A. Gray). These proteins have shown cytotoxicity in human cell lines and have exhibited sensitivity toward solid tumor samples of breast cancer cells [47]. AMPs have also been identified, isolated, and characterized from wheat seeds (*Triticum kiharae* Dorof. et *Migusch*). Among the various sequenced peptides, thionin-like AMPs, including β-purothionin, have been identified. This peptide has demonstrated antimutagenic activity in human RD cells and has shown high efficacy in protecting the DNA of human cells exposed to cadmium chloride, thus highlighting its potential [48]. These findings further underscore the effectiveness of this plant defense strategy in the production of AMPs [124,125].

Transgenic tobacco plants expressing peptides from the thionin family have demonstrated antimicrobial activity, providing resistance against pathogenic fungi (*B. cinerea*), phytopathogenic bacteria (*P. solanacearum*), and insect larvae (*Helicoverpa armigera*) [121]. Furthermore, additional studies have confirmed the antifungal properties of thionin genes when expressed in *Arabidopsis thaliana* plants, resulting in resistance against phytopathogenic fungi (*F. graminearum*) [49].

Thionin genes have been isolated from etiolated barley seeds and seedlings and found to be upregulated in wild-type rice. Consequently, transgenic rice plants expressing elevated levels of thionins from oats in their cell walls exhibited normal growth, even when germinated with phytopathogenic bacteria of the species *Burkholderia plantaii* [35]. This discovery highlights the protective role of thionins in conferring resistance to bacterial infections and underscores the potential of utilizing thionin-based genetic engineering strategies in crop improvement for enhanced disease resistance. In other studies, the expression of rice thionin genes has demonstrated nematocidal activity against detrimental root pathogens in agricultural settings. Specifically, thionins have been shown to reduce susceptibility to nematode infection (*Meloidogyne graminicola*) and colonization by oomycetes (*Pythium graminicola*) [52]. Furthermore, researchers have identified and characterized thionin-like AMPs in *A. thaliana* plants. These peptides have shown in vitro resistance against nematodes (*Heterodera schachtii*) from beet cysts [117].

AMPs derived from the thionin family of *Arabidopsis thaliana* L. have been investigated for their potential applications. AMPs were expressed in the bovine endothelial cell line BVE-E6E7 and exhibited significant antimicrobial activity against various pathogens, including Gram-positive bacteria (*Staphylococcus aureus*), Gram-negative bacteria (*E. coli*), and pathogenic fungi (*Candida albicans*). Additionally, these AMPs demonstrated security to different mammalian cell lines, suggesting their potential use in the treatment of bovine mastitis and other infectious diseases in mammals [36,37]. In another study, a thionin gene from *A. thaliana* L. was expressed in tomato (*Lycopersicon esculentum* Mill.), a member of the Solanaceae family. This expression resulted in antimicrobial activity against the bacterium *Ralstonia solanacearum* and the fungus *F. oxysporum* f. sp. *lycopersici* [122]. When expressed in sweet potato, barley thionin has antimicrobial activity that effectively combats the pathogenic fungus *Ceratocystis fimbriata*. This expression of thionin reduces damage to both leaves and roots, thus promoting the potential use of transgenic foods containing antimicrobial peptides. Such development aims to minimize the dependence on agricultural chemicals [38]. Additionally, when barley thionins are expressed in *Nicotiana benthamiana* Domin., they exhibit remarkable antimicrobial activity. The overexpression of thionins in this plant species has been shown to decrease the susceptibility of the green aphid (*Myzus persicae*), suggesting that the thionin genes possess insecticidal properties [43].

Moreover, antimicrobial activities have been observed for thionins expressed in transgenic onions. In a recent study, the expressed thionin exhibited a 52% inhibitory effect on the germination of *Aspergillus niger* spores, further validating the antifungal properties against pathogenic fungi, which has been consistently reported in previous studies [42].

In conclusion, studies have consistently demonstrated the effectiveness of transgenic plants overexpressing AMP genes in increasing resistance to various pathogenic fungi, bacteria, and insects. These findings emphasize the potential of thionin-based genetic engineering approaches for crop improvement and the development of genetically modified foods with improved disease resistance, thus reducing reliance on agricultural chemicals.

### 2.2. Heveins

Hevein-like peptides are small molecules comprising 29 to 45 amino acids, characterized by a high content of glycine, cysteine, and aromatic amino acids, along with three to five disulfide bonds. These cysteine-rich peptides adopt a compact fold stabilized by multiple disulfide bonds, ensuring exceptional stability. Their three-dimensional structure includes a chitin-binding domain, enabling specific interactions with fungal cell walls by recognizing chitin. The conserved structure of heveins features a β-sheet and cysteine knot motifs, ensuring functional specificity and resistance to degradation. These compounds are found in several monocotyledonous and dicotyledonous plants, for example, in rubber tree latex. These peptides have chitin as their main target, which is useful for studying the interaction of peptides with carbohydrates, which is mainly based on hydrogen bonding and van der Waals forces (Figure 1b) [11,41].

The conformation of hevein-like peptides favors the inhibition of fungal hyphal growth by binding to fungal chitin, protecting plants from a wide variety of fungal pathogens [119]. Hevein-like peptides are of medical and agricultural interest for their association with latex allergy problems and for their antifungal and insecticidal activities. In addition, some hevein-like peptides also have a compact structure that makes them resistant to thermal, chemical, and proteolytic degradation [126].

Furthermore, studies indicate that hevein-like compounds can also act as flocculating agents. Hevein-like peptides were identified in the seeds of moringa species (*Moringa oleifera*), which are members of the Moringaceae family and exhibit coagulation/flocculation activities, demonstrating the highly efficient potential of these peptides [127].

**Figure 3:**
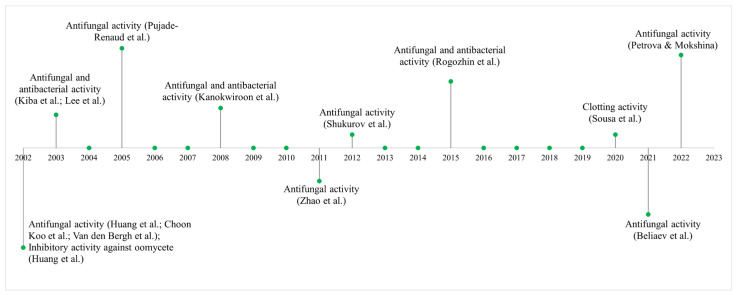
Timeline of scientific papers about main biological activities and gene expression of hevein-like type AMPs from 2002 to 2023 [53,54,55,56,57,58,59,60,61,62,63,64,127,128].

Studies on the biological activities and gene expression of hevein-like AMPs have shown that they exhibit many antimicrobial activities, especially in resistance against phytopathogenic fungi (Figure 3). Hevein-like AMPs were isolated from the bark of the tree (*Euonymus europaeus* L.), which belongs to the Celastraceae family. These peptides showed antifungal activity against phytopathogenic fungi, including *B. cinerea* [53]. In other studies, hevein-like AMPs were also isolated from tree bark (*Eucommia ulmoides* Oliv.) of the Eucommiaceae family and shown to have inhibitory effects on oomycete (*Phytophthora infestans*) and phytopathogenic fungi, *Aculops lycopersicum*, *Verticillium dahliae*, *Gibberella zeae*, *Alternaria nicotianae*, *Fusarium moniliforme*, *F. oxysporum* and *Colletotrichum gossypii* [54].

Studies have shown that hevein-like peptides from *Hevea brasiliensis* Muell. Arg. from the Euphorbiaceae family have antimicrobial activity. The peptides exhibited inhibitory effects on species of pathogenic fungi (*C. albicans, Candida tropicalis,* and *Candida krusei*) and Gram-negative bacteria (*Porphyromonas gingivalis*, *Prevotella intermedia*, *Tannerella forsythia*, *Aggregatibacter actinomycetemcomitans*) [55].

Hevein-like antimicrobial peptides were found in plants of the Moraceae family (*Broussonetia papiyrifera* (L.) L’Hér. ex Vent. and *Morus papirifera* L.). These peptides were isolated, identified, and characterized, and shown to have antifungal effects on *Trichoderma viride* fungi obtained from paper mulberry leaves [56].

Hevein-like peptides were isolated from weed leaves (*Stellaria media* L.) belonging to the Caryophillaceae family. These peptides showed in vitro antimicrobial activities against phytopathogenic fungi (*F. solani, Alternaria alternata, Bipolaris sorokiniana, B. cinerea* and *Aspergillus niger*) and bacteria (*E. coli, P. carotovora, Clavibacter michiganensis* pv. *michiganensis, A. rhizogenes, Bacillus subtilis* and *Pseudomonas syringae* pv. *tomato*) [57].

Other hevein-like peptides were isolated and characterized from *Pharbitis nil* seeds. These peptides were expressed in tobacco plants, conferring resistance against pathogenic fungi and parasites such as the oomycete *Phytophthora parasitica*, the causative agent of black cinnamon disease in plants [58].

Hevein-like peptides have been identified in the leaves of plants (*Wasabia japonica* L.) belonging to the Brassicaceae family. The isolated and characterized peptides exhibit antimicrobial activity when expressed in plants (*N. benthamiana*) of the Solanaceae family, where they confer resistance against pathogenic fungi (*B. cinerea*) and bacteria (*Pseudomonas cichorii, Pseudomonas glumae, Pseudomonas plantaii, A. tumefaciens* and *E. coli*). Furthermore, the peptides inhibited the growth of phytopathogenic fungi (*A. alternata, B. cinerea, F. solani* and *Magnaporte grisea*) and Gram-negative bacteria (*E. coli*) in vitro [59].

Previous studies have shown that hevein-like peptides isolated from *Ipomoea nil* (L.) of the Convolvulaceae family exhibit antifungal activity against a broad spectrum of fungi containing or not containing chitin [60]. Later studies showed that these peptides expressed in tomato (*L. esculentum* Mill.) exhibited antifungal activity, conferring resistance against oomycete (*Phytophthora capsici*) and chitin-containing fungus (*F. oxysporum*) [61].

In other studies, hevein-like genes were isolated from the rubber tree (*H. brasiliensis* Muell. Arg.) and expressed in Nipponbare rice (*Oryza sativa*). Transgenic rice plants exhibit antimicrobial activity against rice blast fungus (*Magnaporthe grisea*), which regulates fungal infections on leaves [62].

Hevein-like peptides isolated from chickweed seeds (*S. media*) showed antimicrobial activity. These peptide genes were expressed in mustard (*A. thaliana* L.) and tobacco (*Nicotiana tabacum* L. cv. *Samsun-NN*) plants resistant to phytopathogenic fungi (*Bipolaris sorokiniana* and *Thielaviopsis basicola*) [63].

Later studies identified hevein-like peptides in common chickweed plants (*S. media* (L.) Vill.) of the Caryophyllaceae family that showed antifungal activity in transgenic plant lines. The peptides were expressed in potatoes (*Solanum tuberosum* L.) of the Solanaceae family and aided in resistance against fungi of the genus *Alternaria* [64].

Hevein-like genes were expressed in tissues of flax (*Linum usitatissimum* L.) of the Linaceae family infected with *F. oxysporum* fungi. Flax-expressing genes from the hevein family exhibited reduced fungal growth, suggesting that these genes confer antifungal activity [128]. In conclusion, these studies revealed the potential of hevein-like peptides as antimicrobial agents in plants, mainly against phytopathogenic fungi and bacteria. These findings are relevant for the development of new plant disease control strategies and alternative antimicrobial options and offer promising prospects for innovative plant protection strategies and potential applications in biotechnology.

### 2.3. Defensins

Defensins are amphipathic molecules of 45 to 54 amino acid residues that contain cationic charges and highly conserved cysteine bonds throughout the family, forming at least four intramolecular disulfide bonds. This conformation allows the formation of three antiparallel β structures and an α-helix that confers high stability. The three-dimensional structure of defensins features an α-helix and an antiparallel triple-stranded β-sheet. The primary structural hallmark of the defensin molecule is the so-called cysteine-stabilized α-helix β-sheet motif (CSαβ), where two cysteine residues separated by a turn in the α-helix are connected to two cysteines located at a single amino acid within the third β-strand (Figure 1c) [9].

Plant defensins are classified into two groups based on the structure of the precursor: class I (absence of the C-terminal prodomain), which provides the first line of defense against the invasion of phytopathogens into the extracellular space, and class II (presence of the prodomain C-terminal), which prevents the phytotoxic effects of defensin against other host cells. In addition, many defensins have been isolated from seeds, but they have also been identified in other tissues, such as leaves, fruits, flowers, and roots [129,130,131,132].

The primary structure of plant defensins is quite diverse, with the exception of the eight cysteine residues, which reflects the different in vitro biological activities already described. These include a broad spectrum of activities, such as antibacterial, antifungal, insecticidal, antiviral, and anticancer activities; the inhibition of trypsin activity; and the production of α-amylases in some insects and fungi. In addition, they have antiproliferative effects, acting as ion channel inhibitors and pathogen protein synthesis inhibitors; inducing the formation of Reactive Oxygen Species (ROS); promoting apoptosis in yeast and other fungal species; and promoting wound healing, proliferation control, and chemotaxis. Its biological functions are relevant, and it has therapeutic potential in combination with medicinal and agricultural treatments [133,134,135,136].

The mechanism of action of defensins may be subject to electrostatic interactions, preferentially related to negatively charged structures of the cell membrane of pathogens, resulting in increased membrane permeability and cell leakage followed by cell death. Some studies point to an alternative mechanism of action that does not damage the cell membrane of pathogens. In this case, these defense peptides are internalized in the intracellular environment, leading to high ionic penetrability via reactions with membrane phospholipids [137].

**Figure 4:**
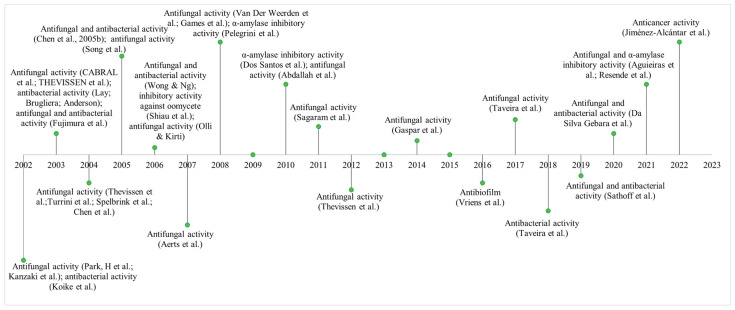
Timeline of scientific papers about main biological activities and gene expression of defensin-type AMPs from 2002 to 2023 [65,66,68,69,70,71,72,73,74,131,132,138,139,140,141,142,143,144,145,146,147,148,149,150,151,152,153,154,155,156,157,158].

The biological activities and gene expression of plant defensins have been extensively studied over the years due to their range of bioactive properties against pathogens and for the treatment of human diseases (Figure 4). For example, defensins isolated from the Mexican avocado (*Persea americana* var. *drymifolia* Mill.) of the Lauraceae family are cytotoxic to the Jurkat lineage of acute lymphocytic leukemia cells. In the present study, defensins inhibited cell viability and induced cell death through caspase-dependent apoptosis. In addition, inducing increased ROS production and a decrease in the mitochondrial membrane potential presents great potential for studies of anticancer activity [138].

Defensins isolated from the dahlia plant (*Dahlia merckii* Lehm.) of the Asteraceae family have membrane interactions with sphingolipids isolated from *Saccharomyces cerevisiae*. In the present study, the interaction of defensin with the membrane increased in the presence of ergosterol, and these data suggest that this peptide has antifungal activity [139]. In other studies, this defensin was expressed in eggplant plants (*Solanum melongena* L.) of the Solanaceae family, which were resistant to the pathogenic fungi *B. cinerea* and *Verticillium albo-atrum* [140].

In studies carried out by [141], defensin peptides found in the plant *Medicago truncatula* Gaertn., which belongs to the Fabaceae family, exhibited growth-inhibitory activity against Gram-negative bacteria (*E. coli*, *P. syringae* pv. *syringae*, *Sinorhizobium meliloti,* and *Xanthomonas alfalfae* subsp. *alfafa*) and filamentous fungi (*Phoma medicaginis* and *F. solani*).

Defensins isolated from ornamental tobacco flowers (*Nicotiana alata* Link and Otto) of the Solanaceae family have antifungal and antibacterial effects on filamentous fungi and bacteria, inhibiting the growth of *F. oxysporum* and *B. cinerea* in vitro [142,143]. These results are similar to those of studies in which defensins were isolated from buckwheat seeds (*Fagopyrum esculentum* Moench.) that also showed antibacterial activity conferring resistance against Gram-positive (*Clavibacter michiganensis* and *Curtobacterium flaccumfaciens*) and Gram-negative (*P. carotovora*, *A. rhizogenes* and *A. radiobacter*) strains and antifungal activity against filamentous (*F. oxysporum*) and nonfilamentous (*Geotrichum candidum*) fungi [144].

Furthermore, defensin-like AMPs were isolated from alfalfa seeds (*Medicago sativa* L.) of the Fabaceae family and characterized. The defensins of this leguminous plant showed in vitro antifungal activity, conferring resistance to fungi of the *F. graminearum* species [145].

Defensin-like AMPs have also been extracted from adzuki bean (*V. angularis* (Willd.) Ohwi and H. Ohashi) seeds of the Fabaceae family, showing antimicrobial activity. The defensin was purified and characterized, and it was found that it exhibited antifungal and antibacterial activity, conferring inhibition of the growth of pathogenic fungi (*F. oxysporum*, *F. oxysporum,* F. sp. *pisi* and *Trichophyton rubrum*), Gram-positive bacteria (*Staphylococcus epidermidis* and *Bacillus cereus*) and Gram-negative bacteria (*Xanthomonas campestris* pv. *vesicatoria*, *Salmonella typhimurium*, *Salmonella enteritidis*, *E. coli*, *P. carotovora* pv. *carotovora*, *Proteus vulgaris* and *P. syringae* pv. *syringae*) [146].

Defensin-like AMPs isolated from the lima bean (*Phaseolus limensis* L.) seed of the Fabaceae family have already been identified. AMPs showed antifungal and antibacterial activity, inhibiting the growth of *B. cinerea*, *F. oxysporum*, and *Mycosphaerella arachidicola* [147]. In radish seeds (*Raphanus sativus*), AMPs from the defensin family that exhibited antifungal activity were identified. In the present study, defensin inhibited the growth of the yeast species *C. albicans* and *Pichia pastoris* [148].

In other studies, defensins isolated from radish seeds (*R. sativus* L.) that interact with glycosylceramides were shown to have antifungal activity. Defensins exhibit fungal cell membrane permeabilization activity, fungal cell wall stress, and reactive oxygen species production in *C. albicans,* conferring growth-inhibitory activity against fungal cells [149,150]. In later studies, these defensins induced apoptosis and concomitantly triggered the activation of caspases or caspase-like proteases in *C. albicans* [159]. Furthermore, the potential antibiofilm activity of radish defensins expressed in *P. pastoris* in combination with caspofungin against *C. albicans* biofilms was analyzed [151].

Plant defensins isolated from Chinese cabbage seeds (*Brassica campestris* L. ssp. *pekinensis*) were expressed in *E. coli* and showed antimicrobial activity. These overexpressed peptides exhibited antifungal activity, conferring resistance against the phytopathogenic fungi *A. solani*, *Neurospora crassa*, *Phytophthora parasitica,* and *F. oxysporum* [152]. Plant defensins overexpressed in rice (*Oryza sativa* cv. *Sasanishiki*) also exhibit antifungal activity, conferring resistance to phytopathogenic fungi (*Magnaporthe grisea*) in transgenic rice [153].

Defensin-like AMPs were identified from a cDNA clone isolated from winter wheat tissue (*Triticum aestivum* L.) that exhibited antibacterial activity. Peptides expressed in winter wheat sprouts inhibited the growth of the phytopathogenic bacterium *P. cichorii* [154].

Plant defensins were isolated from mung bean or mung bean leguminous plants (*Vigna radiata* (L.) Wilczek) of the Fabaceae family, known as moyashi. Plant defensins expressed in artificial seeds exhibited in vitro insecticidal activity against the beetle *Callosobruchus chinensis*, inhibiting the growth of bruchid larvae [160]. In later studies, mung bean defensin was expressed in *P. pastoris* and showed antifungal activity against the fungi *F. oxysporum*, *Pyricularia oryzae*, *Rhizoctonia solani,* and *Trichophyton rubrum* [155].

Defensins isolated from cowpea or black-eyed pea (*V. unguiculata*) seeds were expressed in *E. coli*. These AMPs showed enzymatic inhibitory effects and inhibited α-amylases in insect pests (*Acanthoscelides obtectus* and *Zabrotes subfasciatus*), highlighting their potential in the development of transgenic plants to control insect pests [65,66].

Antimicrobial peptides of the defensin family were isolated from common bean seeds (*Phaseolus vulgaris* cv. Pérola) and characterized. From messenger RNA (mRNA) of *P. vulgaris seeds*, complementary DNA (cDNA) was synthesized by reverse transcriptase–polymerase chain reaction (RT–PCR) and cloned; this cDNA encoded a peptide belonging to the defensin family. These defensins inhibited the growth of yeasts *(C. albicans*, *Candida parapsilosis*, *C. tropicalis*, *Candida guilliermondii*, *Kluyveromyces marxiannus,* and *S. cerevisiae*) and inhibited the growth of phytopathogenic fungi (*F. oxysporum*, *F. solani*, *F. lateritium,* and *Rizoctonia solani*) [67].

Recombinant defensins isolated from Mexican radish seeds (*Pachyrhizus erosus* (L.) Urb.) showed antifungal activity. Studies have shown that defensins expressed in *P. pastoris* exhibit growth-inhibitory effects on various pathogenic fungi (*F. oxysporum* f. sp. *vasinfectum*, *Verticillium dahliae*, *Aspergillus flavus*, *Penicillium* spp., *Colletotrichum gloeosporioides*, *B. cinerea*, *Bipolaris maydis*, *A. niger*, *F. oxysporum* f. sp. *lycopersici* and *Rhizopus stolonifer*) [68].

Recombinant AMPs of the defensin family were identified and isolated from Peruvian maca *(Lepidium meyenii*) of the Brassicaceae family and showed antimicrobial activity. The recombinant peptide exhibited in vitro growth inhibition activity against *P. infestans* [69]. Recombinant pea (*Pisum sativum*) defensins expressed in yeast (*P. pastoris*) showed antifungal activity, conferring resistance against *Aspergillus niger* [70].

Recombinant defensins were isolated from fenugreek plants (*Trigonella foenum-graecum*), and when expressed in *E. coli,* they exhibited antifungal activity against phytopathogenic fungi (*Rhizoctonia solani* and *Phaeoisariopsis personata*) [71]. Furthermore, AMPs from the defensin family isolated from the alfalfa (*M. sativa* L.) family Fabaceae when expressed in transgenic tomato (*Lycopersicum esculentum* Mill.) showed antifungal activity. Transgenic tomato plants were shown to be resistant to pathogenic fungi (*F. oxysporum* f. sp. *lycopersici*) [72]. Additionally, plant defensin-like genes were isolated from *Nicotiana alata* and shown to exhibit antifungal activity when expressed in transgenic cotton. These defensin-like compounds showed antifungal activity against *Fusarium oxysporum* e *Verticillium dahliae* wilt [73].

Some studies have shown that the biological activity of some plant defensins is related to an amino acid region located between the β2 and β3 strands, called the γ-core region. De Samblanx et al., 1997 showed that alterations in the amino acid residues present in the γ-core reduced the biological activity of *Rs*AFP2 (a *Raphanus sativus* defensin). Another study showed that the γ-core region is a determinant of the antifungal activity of *Ms*Def1 and *Mt*Def4 (defensins from *M. sativa* and *M. truncatula*, respectively) [74]. Overall, these findings underscore the vast potential of plant defensins as resources for developing novel strategies against infectious diseases and plant protection against pathogens and for potential therapeutic applications in cancer research and human disease treatment. The exploration of plant defensins has led to the identification of their diverse biological activities, holding promise for future advancements in biotechnology and medicine.

### 2.4. Lipid Transfer Proteins

Lipid transfer proteins (LTPs) were isolated for the first time from potato tubers [75]. These cationic molecules are highly expressed in most tissues but are absent in most groups of basal plants. These peptides consist of approximately 100 amino acid residues and are relatively larger in size than other AMPs, such as defensins [41,76]. LTPs have a three-dimensional structure that enables them to interact with lipids and mediate their transport between cellular membranes. Many LTPs exhibit a compact, barrel-like structure, often containing hydrophobic residues and a β-barrel or α-helix motif, stabilizing the protein while allowing flexibility in lipid binding and transport, forming a binding cavity that accommodates lipids for transfer (Figure 1d) [77].

LTPs are divided into two major subfamilies according to their molecular size: LTP1 and LTP2. However, LTPs have recently been grouped according to the position of the conserved intron, the identity of the amino acid sequence, and the spacing between cysteine residues; there are five main types of LTPs: LTP1, LTP2, LTPc, LTPd, and LTPg. Its C-terminal sequence allows the integration of LTPs on the extracellular side of the membrane [77,78,119].

In general, LTPs perform activities that mediate the transfer of lipids across the cytoplasm; that is, they supply lipids at membrane contact sites, which include fatty acids, phospholipids, prostaglandin B2, lysate derivatives, and acyl-coenzyme A or sterols [79]. In this way, they can stimulate the extraction of lipids from the membrane, mobilization of lipids into the aqueous cytoplasm, and reinsertion of lipids into a different membrane. LTPs can inhibit the growth of fungi and some bacterial pathogens and are also involved in the plant defense system [77].

Several studies have shown that LTPs play an important role in plant defense. The LTPs identified in coffee species (*Coffea canephora* L.) belonging to the Rubiaceae family exhibited high antimicrobial activity. The isolated peptides inhibited the growth of phytopathogenic fungi (*Colletotrichum lindemuthianum*, *C. gloeosporioides*, *Fusarium solani*, *Fusarium lateritium,* and *Colletotrichum* sp.) and Gram-negative bacteria (*Xanthomonas euvesicatoria*), in addition to increasing the membrane permeability and inducing ROS in all the fungi tested [80]. Currently, studies on LTPs show that these peptides have other therapeutic potential, such as cytotoxic and antiproliferative effects on tumor-derived cells, antiviral activity, inhibition of digestive enzymes, and antinociceptive activity [81].

**Figure 5:**
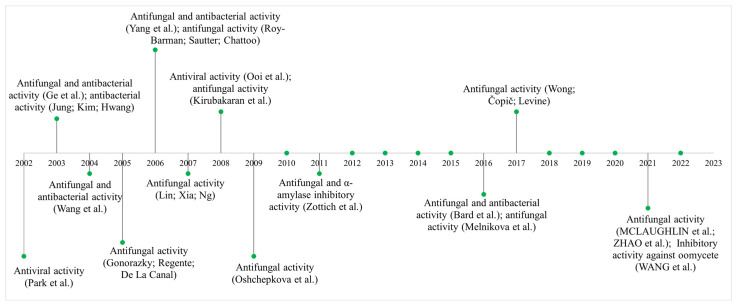
Timeline of scientific papers about main biological activities and gene expression of LTP-type AMPs from 2002 to 2023 [77,80,82,83,84,85,86,87,88,89,90,91,92,93,94,95,96,97].

Studies have shown that LTP-type AMPs have great therapeutic potential due to their antifungal, antibacterial, and antiviral properties (Figure 5). LTP-like AMPs have been identified from pepper leaf cDNA (*Capsicum annuum* L. cv. Bugang). The expression of LTPs was induced when the leaves were infected with TMV. In other studies, LTPs were expressed in response to pepper leaf infection by pathogenic bacteria (*X. campestris* pv. *vesicatoria*), suggesting that LTPs play an important role in plant defense against viral and bacterial pathogens [82,83].

Likewise, LTPs isolated, purified, and identified from rice leaves showed antimicrobial activity in vitro. LTPs exhibit antifungal and antibacterial activity by inhibiting the germination of fungal spores (*P. oryzae*) and the growth of Gram-negative bacteria (*Xanthomonas oryzae*) [84].

Furthermore, nonspecific lipid transfer protein (nsLTP) AMPs were isolated from mung bean seeds (*Phaseolus mungo*) and exhibited antifungal and antibacterial activity. LTPs confer resistance against pathogenic fungi (*F. solani*, *F. oxysporum*, *Pythium aphanidermatum,* and *Sclerotium rolfsii*) and Gram-positive bacteria (*S. aureus*) [85].

The LTPs identified in potato plants were induced by an oomycete (*Ph. infestans*) and phytohormones (ABA, abscisic acid, salicylic acid, and jasmonic acid), providing plant resistance against the pathogen *Ph. infestans* correlated with the amount of ROS generated during plant defense responses [86]. The nsLTP-like AMPs isolated from motherwort seeds (*Leonurus japonicus* Houtt.) showed in vitro antifungal and antibacterial activity. LTPs inhibited the growth of filamentous fungi (*F. oxysporum, Alternaria brassicae*, *Bipolaris maydis,* and *Rhizoctonia cerealis*), yeasts (*S. cerevisiae*), and Gram-positive bacteria (*Bacillus subtilis*) [87].

LTP-like AMPs were isolated from seeds of *Brassica campestris* L. and showed antifungal activity. LTPs exhibited inhibitory effects on the mycelial growth of pathogenic fungi (*F. oxysporum* and *M. arachidicola*) [88]. LTPs isolated from sunflower seeds (*Helianthus annuus*) have been previously described to have high antifungal activity (*F. solani* f. sp. *eumartii*). Later studies demonstrated that these LTPs were able to inhibit the germination of spores from other phytopathogenic fungi, such as *A. alternata* [89].

LTP-like AMPs isolated from daffodils (*Narcissus tazetta* var. *chinensis* L.) exhibited in vitro antiviral activity. These LTPs were able to significantly inhibit plaque formation via respiratory syncytial virus (RSV) and cytopathic effects via the *influenza A* (H1N1) virus [90].

LTPs isolated from the seeds of black cumin flowers (*N. sativa* L.) exhibited antifungal activity. These AMPs inhibit the growth of phytopathogenic fungi (*P. debaryanum*) and oomycetes (*Ph. infestans*) [91]. Likewise, LTP-like AMPs have also been isolated and identified from coffee seeds (*Coffea canephora*). These LTPs showed antifungal activity against yeasts (*C. albicans*) in addition to conferring morphological alterations to *C. tropicalis* and inhibiting the activity of α-amylase in mammals in vitro [92].

The nsLTP-like AMPs isolated from transgenic wheat were expressed in *P. pastoris* and exhibited growth-inhibitory effects on a pathogenic fungus (*F. graminearum*). In addition, nsLTPs suppressed ROS production in plants [93]. Other LTP-like AMPs isolated from wheat and expressed in *E. coli* also exhibited antifungal activity against pathogenic fungi (*Alternaria* sp., *Rhizoctonia solani*, *Curvularia lunata*, *Bipolaris oryzae*, *Cylindrocladium scoparium*, *B. cinerea* and *Sarocladium oryzae*) [94].

LTP-like AMPs isolated from dill (*Anethum graveolens* L.) when expressed in *E. coli* bacteria showed weak antifungal activity. However, they inhibited spore germination, delayed the elongation of *A. niger* hyphae, and exhibited binding with phytohormones [95].

LTPs from onions (*Allium cepa* L.) were expressed in the ears of transgenic wheat plants and showed antifungal activity. LTPs confer resistance to phytopathogenic fungi (*Blumeria graminis* f. sp. *tritici* and *Neovossia indica*) in transgenic rice [96]. In other studies, the LTPs expressed in transgenic wheat (*T. aestivum*) conferred plant resistance to the leaf rust pathogen (*Puccinia triticina* Erikss.) and induced the plant to produce ROS [97].

In conclusion, LTPs are found in various natural food sources, including pepper leaves, rice leaves, mung bean seeds, and potatoes. These plants exhibit antifungal, antibacterial, and antiviral activities and play an important role in enhancing plant defense against pathogens. Moreover, LTPs possess diverse antimicrobial properties and hold promise as potential therapeutic agents in medicine and agriculture.

### 2.5. Cyclotides

Cyclotides are plant-derived cyclic antimicrobial peptides that occur in a wide variety of families, including Rubiaceae (coffee family), Violaceae (violet family), Fabaceae (legumes), Solanaceae (belladonna family and peppers of the genus *Capsicum*) and Cucurbitaceae (cucurbits) [161,162]. These cyclic peptides typically comprise 30 amino acids and can be found in a wide variety of plant tissues, including leaves, flowers, stems, and roots.

Cyclotides are characterized by a cyclic backbone, which is stabilized by three disulfide bonds that form a cysteine knot that makes them resistant to proteolysis and thermal denaturation. Structurally, cyclotides consist of a fold forming a cyclic structure that is often surrounded by hydrophobic residues (Figure 1e) [162,163].

In the 1970s, the first cyclotide, called kalata B1, found in the African tropical plant *Oldenlandia affinis* was described and was used by local populations as a medicine to accelerate “childbirth”. In 1995, this fungus was characterized, and since then, other cyclic peptides have been isolated from plant biomass and characterized [163].

Cyclotides have a cyclic cystine knot (CCK), a structure with six conserved cysteines and composed of two disulfide bonds (CysI-CysIV and CysII-CysV) combined with a third disulfide bond in the β strand (CysIII-CysVI). This conformation results in a unique protein fold. The distorted triple strand together with the structural complexity of “head-tail” cyclization is responsible for exceptional biological activities, as the peptide exhibits resistance to enzymatic and proteolytic breakdown, high thermal stability, and chemical denaturation [164]. These cyclic peptides have diverse biological activities, including hemolytic, neurotensin antagonist, anti-HIV, antimicrobial, protease inhibitor, insecticidal, antitumor, antifouling, nematicidal, molluskicidal, immunosuppressive, and inhibitory activities of the enzyme prolyl oligopeptidase [165,166,167].

Cyclotides can be classified into three subfamilies: Möbius, bracelet, and trypsin inhibitors. Möbius cyclotides can be found in Rubiaceae family plants such as *O. affinis.* The trait structure of Möbius is the presence of a Pro residue in the cis-peptide bond in loop 5, which induces a 180° twist in the peptide backbone. Bracelet cyclotides, such as *V. odorata,* are found in the Violaceae family and are distinguished by the absence of Pro residues, although the möbius and bracelet subfamilies are highly hydrophobic and have conserved residues that enable interactions in cell membrane binding. The trypsin inhibitor cyclotides can be found in Cucurbitaceae plants, such as *M. cochinchinensis*. Furthermore, these proteins have low sequence similarity with other subfamilies but possess the characteristic CCK motif of cyclotides [168,169].

Recent phylogenetic studies have sought to understand the evolutionary history of cyclotide precursor diversity among Solanaceae plant species. Researchers have revealed new cyclotide precursor genes in the Solanaceae family, unlike what has been found to date. The identification of cyclotide precursor genes helps in exploring their evolutionary link with other families, in addition to investigating their biological activities, enabling the development of new therapies [170].

**Figure 6:**
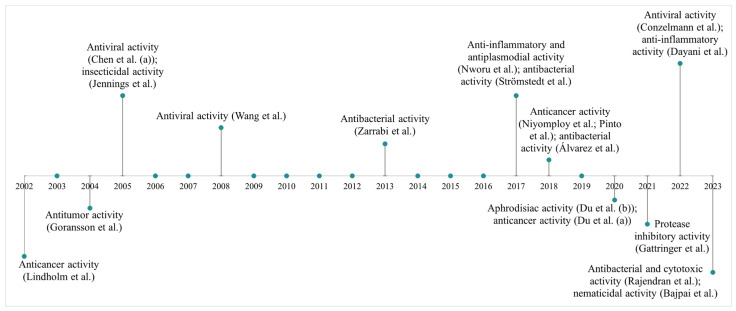
Timeline of scientific papers about main biological activities and gene expression of cyclotide types of AMPs from 2002 to 2023 [98,99,100,101,102,103,104,105,106,107,108,109,110,111,112,113,115,116].

Cyclotides have been intensely studied due to their bioactive properties of medical interest (Figure 6). These compounds exhibit a range of biological activities that make them promising for development as therapies against cancer and infectious diseases. [171]. Previous studies have shown that AMPs from the cyclotide family isolated from field pansy (*Viola arvensis* Murr.) and wood violet (*Viola odorata* L.) exhibit antitumor and cytotoxic effects on human tumor cell lines. These studies were important for identifying the anticancer effects of cyclotides and demonstrating their potential in the development of antitumor drugs based on AMPs [98,99].

In other studies, on plant AMPs, cyclotides isolated from the Indian medicinal herb *Hybanthus enneaspermus* (L.) F. Muell.) of the Violaceae family with in vivo aphrodisiac properties were identified. Studies have suggested that cyclotides can increase libido in addition to facilitating erectile function in male rats [100]. Furthermore, the cyclotides of this indigenous plant also showed anticancer activity. These AMPs have been shown to induce membrane blistering and cell necrosis [101]. This finding suggested that cyclotides have great potential for anticancer activity, as already reported in other studies [102,103].

Recent research has shown that cyclotide-containing extracts from wild pansy (*Viola tricolor*) exhibit in vitro inhibitory activity against human immunodeficiency virus (HIV) [104]. Cyclotides exhibited inhibitory effects on HIV by reducing the cytopathic effects of HIV infection [105]. These findings are similar to those of other previous studies in which cyclotide-like AMPs were isolated from the Chinese medicinal herb *Viola yedoensis* Makino which showed antimicrobial activity. Plant extracts containing cyclotides exhibited anti-HIV activity according to in vitro MTT assays [106].

In recent studies, cyclotides isolated from wood violet were shown to significantly reduce inflammation and demyelination in mice and alleviate neurological deficits in an experimental model of experimental autoimmune encephalomyelitis (EAE) [107]. In other studies, cyclotides isolated from *O. affinis* were investigated for their suppressive anti-inflammatory and antiplasmodial activities against *Plasmodium berghei* in rodents, which significantly inhibited acute and chronic inflammation in rodent models [108]. In addition, these AMPs have also been identified in plants of the genus *Allexis* of the Violaceae family that exhibit protease inhibitory activity, conferring inhibition of human prolyl oligopeptidase activity [109].

Studies show that cyclotides are particularly potent against Gram-negative bacteria such as *Pseudomonas aeruginosa*, *Aeromonas salmonicida*, *Vibrio ordalii*, *Vibrio anguillaru*, *Flavobacterium psychrophilum* and *E. coli* [110,111]. Plant pathogenic bacteria are more susceptible than human pathogenic bacteria. However, previous studies have shown that cyclotides isolated from wood violet plants exhibit antibacterial activity by inhibiting the growth of Gram-positive bacteria (*S. aureus*) [112]. In recent studies, cyclotides isolated from the leaves and petioles of *Geophila repens* of the family Rubiaceae demonstrated antibacterial and cytotoxic activity. AMPs of the cyclotide family showed antibacterial activity against *E. coli* and cytotoxic activity in the U-937 human lymphoma cell line [113].

Cyclotides exhibit a wide range of bioactive activities, including insecticidal activities. AMPs exhibit insecticidal activity, reducing the growth and development of moth larvae (*Helicoverpa armigera*) [114,115]. In a recent study, cyclotides isolated from extracts of *O. affinis, Clitoria ternatea*, *Viola odorata,* and *Hybanthus enneaspermus* showed nematocidal effects on *Caenorhabditis elegans* larvae. Cyclotides can cause death and damage, such as membrane disruption and toxicity, in *Caenorhabditis elegans*, possibly leading to larval death [116].

In conclusion, cyclotides are bioactive peptides with diverse therapeutic potential. They exhibit promise as cancer therapies, display antiviral and antibacterial properties, and show activity against insects and nematodes. Additionally, cyclotides have been shown to have aphrodisiac and anti-inflammatory effects. The biological activity of cyclotides highlights their potential applications in medicine and agriculture, suggesting that they are promising therapeutic agents.

## 3. AMPs from the Solanaceae Family

The Solanaceae family is home to a group of important plants, mainly in the Brazilian culture and economy, as potatoes, tomatoes, eggplant, and peppers, which belong to this family, have typically been present in the country’s cuisine for decades. Among the most studied plants, those of the genus *Capsicum* can be highlighted due to their biological activities against pathogens, enhancing their therapeutic use [172].

Recent studies have shown that several groups of antimicrobial peptides of vegetal origin present in the Solanaceae family act as the main components in defense mechanisms against microorganisms. For example, [173] extracted protein from potato (*Solanum tuberosum* L.) with the ability to inhibit the growth of oomycetes (*P. infestans*) and bacteria (*S. aureus*), suggesting new possibilities for the use of potato protein. AMPs present in the Solanaceae family could be used for the development of transgenic plants resistant to pathogens for crop improvement.

AMPs extracted from potatoes exhibit broad-spectrum antimicrobial activity both in vitro and in vivo, highlighting their important role in the defense system of plants. For example, potato Snakina-1 (SN1) is active in vitro against bacteria, fungi, yeast, and even animal/human pathogens, as transgenic potato plants overexpressing SN1 exhibited growth-inhibitory activity against bacterial and fungal pathogens. Furthermore, it has been shown to confer in vivo protection against commercially relevant pathogens in potato, wheat, and lettuce plants. In this way, SN1 could be a candidate for pharmaceutical or agricultural biotechnology applications by acting as a preservative agent for the food, pharmaceutical or cosmetic industry [174].

In other studies, defensin-like AMPs and snakins were isolated from tomato plants (*Solanum lycopersicum* L.). The peptides exhibited antimicrobial activity against yeast (*Cryptococcus neoformans* and *C. albicans*), Gram-positive (*Clavibacter michiganensis*), and Gram-negative (*P. savastanoi* and *P. carotovorum*) bacteria. In this way, it becomes possible to use these peptides as new antimicrobial agents to control bacterial wilt and canker in tomato plants, in addition to being promising in the development of pharmaceutical products [175].

In the Solanaceae family, many antimicrobial peptides that have great potential in the development of products of medical and agricultural interest have already been identified and characterized. Among the classes of AMPs isolated from this family are defensins, LTPs, thionins, and protease inhibitors. Normally, these AMPs are produced in response to injury and inoculation by pathogens [176].

**Figure 7:**
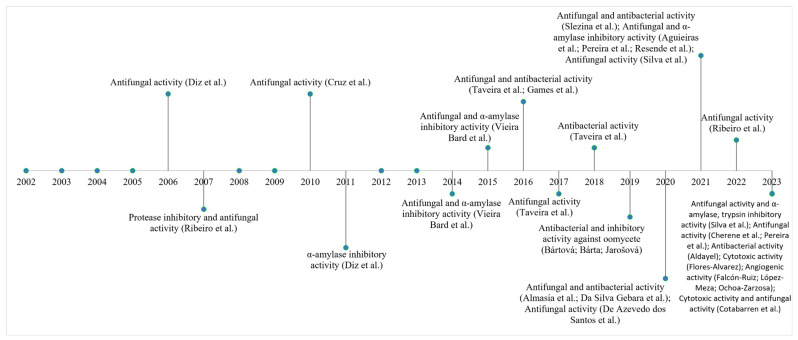
Timeline of scientific papers about main biological activities and gene expression found in plants of the genus *Capsicum* for the period from 2002 to 2023 [123,131,132,156,157,158,173,174,175,177,178,179,180,181,182,183,184,185,186,187,188,189,190,191,192,193].

Studies on plants of the genus *Capsicum* of the Solanaceae family have shown that several AMPs have already been identified and characterized, and most of them have antifungal and enzyme inhibition activities (Figure 7). For example, LTPs were identified and sequenced in seeds of peppers of the species *C. annuum* L. In these studies, the characterization, immunolocalization, and identification of in vitro mammalian α-amylase inhibitory activity and antifungal activity of LTPs were performed against phytopathogenic fungi *(F. oxysporum* and *C. lindemuthianum*) and yeasts *(S. cerevisiae*, *Pichia membranifaciens*, *C. tropicalis* and *C. albicans*) [177]. Likewise, in other studies, these LTP-like AMPs have been shown to have fungicidal effects on *C. albicans*, *S. cerevisiae,* and *Schizosaccharomyces pombe* in addition to causing morphological changes in yeasts [178].

In other studies, defensin-like peptides capable of inhibiting the activity of different α-amylase enzymes were identified and characterized in *Capsicum chinense* fruits and yeasts of the *Candida* genus, revealing high antifungal potential [131]. In addition, [179] isolated peptides from *C. chinense* fruits that exhibit inhibitory effects on the growth of phytopathogenic fungi, in addition to causing membrane permeabilization, induction of endogenous ROS, activation of caspases, and functional collapse of mitochondria in fungi of the genus *Fusarium*.

Thionin-like AMPs were identified in the genus *Capsicum* highlighting the relevance of research on this genus. In these studies, several aspects of the mechanism of action of these thionin-like peptides were described, and antimicrobial activity against yeast and bacterial species was assayed. The peptides inhibited the growth of the yeasts *S. cerevisiae*, *C. albicans,* and *C. tropicalis*, inducing alterations in the membranes and permeabilization, in addition to causing a reduction in the growth of the bacterial species *E. coli* and *P. aeruginosa* [123].

Recent studies have shown that defensin-like AMPs are isolated from the seedless fruits of *C. chinense* Jacq. exhibit antifungal activity against yeast species (*C. albicans* and *C. tropicalis*) [180]. These studies corroborate previous studies, such as Da Silva Gebara et al. (2020), who identified defensin-like AMPs in pepper fruits (*C. annuum* L.) that exhibit growth-inhibitory effects on fungal species (*C. albicans*, *Candida parapsilosis*, *Candida buinensis,* and *C. tropicalis*) and pathogenic bacteria (*Mycobacterium tuberculosis*). Furthermore, AMPs induce ROS production and fungal cell membrane permeabilization [156].

Thionin-like cationic peptides (CaThi) were also isolated and characterized from fruits of *C. annuum,* and when combined with fluconazole, it was possible to identify antimicrobial activities. In the present study, CaThi showed antifungal activity against species of the genus *Fusarium,* resulting in the total death of *F. solani* by apoptosis when CaThi was combined with fluconazole [157]. In other studies, CaThi, in addition to having antifungal effects on yeast (*C. tropicalis*) and filamentous fungi, was shown to also exhibit antimicrobial activity against bacteria, inducing the activation of caspases and ROS. Furthermore, this peptide promotes an imbalance in pH homeostasis, highlighting its role in modulating H+ transport systems [158].

In other studies of the genus *Capsicum* peptides that inhibit enzymes and proteins extracted from the leaves and roots of pepper plants (*C. annuum* L.) were found. The extracts of leaves and roots inhibited trypsin and α-amylase activity and showed antifungal effects on *Colletotrichum scovillei* [181]. Previous studies have also identified proteinase inhibitor-like AMPs and 2S albumin from pepper seeds that inhibited the growth of yeasts (*S. cerevisiae*, *C. albicans*, *C. parapsilosis*, *C. tropicalis*, *C. guilliermondii*, *Pichia membranifaciens,* and *K. marxiannus*) [182].

The protease inhibitors identified in the genus *Capsicum* were extracted from *C. chinense* Jack. seeds and showed antifungal activities against filamentous fungi (*C. gloeosporioides*, *C. lindemuthianum, F. oxysporum,* and *F. solani*), causing mainly membrane permeability and oxidative stress. In addition, they also reported that these peptides are bifunctional inhibitors that inhibit trypsin activity and *Tenebrio molitor* larval α-amylase activity [183]. Recent research has shown that AMPs isolated from seeds of pepper (*C. chinense* Jacq.) exhibit antifungal activity against phytopathogenic fungi. Peptide-type serinic protease inhibitors inhibited the growth of the fungal species *C. lindemuthianum* and *F. oxysporum,* causing damage to fungal cells. Furthermore, these peptides have been shown to inhibit trypsin, human salivary α-amylase, and *T. molitor* larval α-amylase [184].

Protease inhibitor-like AMPs were identified in sweet pepper fruits (*C. chinense*) and shown to have antifungal activity and enzymatic inhibition. In addition to inhibiting the growth of phytopathogenic fungi (*C. lindemuthianum*, *F. lateritium*, *F. solani* and *F. oxysporum*), AMPs have been shown to inhibit trypsin and α-amylase activities [132]. In another study, AMPs isolated from pepper seeds (*Capsicum baccatum*) inhibited mammalian and insect α-amylase (*Callosobruchus maculatus*) in addition to inhibiting the growth of pathogenic fungi (*S. cerevisiae*, *C. albicans*, *C. tropicalis,* and *K. marxiannus*) [185].

In other studies, serine protease inhibitor-type AMPs isolated from *C. annuum* seeds were identified; these AMPs exhibited antifungal activity and growth inhibition in yeasts (*S. cerevisiae*, *C. albicans*, *C. tropicalis,* and *K. marxianus*), in addition to demonstrating inhibitory activity against human and insect salivary α-amylases (*C. maculatus*) [186].

Hevein-like AMPs extracted from bell pepper leaves (*C. annuum* L. Magali R) showed in vitro antibacterial activity, conferring resistance against Gram-positive bacteria (*Clavibacter michiganensis* ssp. *michiganensis*), Gram-negative bacteria (*Ralstonia solanacearum*, and *P. carotovora*) and phytopathogenic fungi (*Alternaria solani*). In addition, other classes of peptides that have been detected are involved in plant defense against phytopathogens [187].

In a recent study, defensins isolated from Habanero Chile pepper (*C. chinense* L.) were shown to be effective against cancer cells. The peptides were cytotoxic to K562 leukemia cells by modifying the mitochondrial membrane potential and amount of intracellular calcium. Furthermore, these peptides induce caspase-independent apoptosis in cancer cells, suggesting that therapeutic alternatives are promising [188]. In addition, defensin-like compounds isolated from Habanero peppers (*C. chinense* L.) and avocado (*Persea americana*) inhibited the proliferation and migration of cells stimulated by vascular endothelial growth factor. These studies showed that defensin-like peptides could be potential angiogenic modulators [189].

Recent research has shown that protease inhibitors, defensin-like proteins, and lipid transporter proteins isolated from leaf peppers (*C. annuum* L.) exhibit antimicrobial activity against the yeasts *C. albicans*, *C. tropicalis*, *C. parapsilosis,* and *C. buinensis*. Peptides have various effects on yeasts, such as growth inhibition and morphological and physiological changes. These studies suggest that these peptides have the potential to be antimicrobial agents [190]. In another study, AMPs identified and isolated from the leaves and roots of peppers (*C. annuum* L.) exhibited antifungal activity against filamentous fungi. Proteinase inhibitor peptides inhibited fungal growth in the species *C. scovillei* by causing cell membrane permeabilization, endogenous reactive oxygen species induction, and mitochondrial activity reduction [191].

In other studies, synergistic effects of the aqueous extract of bell pepper (*C. annuum*) and chitosan, which exhibit antibacterial activity, were identified. In that study, the combination of chitosan and bell pepper extract demonstrated antibacterial activity against *S. aureus*, *P. aeruginosa*, and *S. typhimurium*, causing significant damage to the bacterial membrane potential [192]. In addition, trypsin protease inhibitors were isolated from yellow bell pepper seeds (*C. annuum* L.) and showed potent antifungal activity against pathogenic fungi. These inhibitors exhibited low cytotoxic activity in human cells and strong antifungal activity in vitro against *C. albicans* and were able to inhibit α-1,4-glucosidase [193].

In conclusion, a diverse range of AMPs has been identified within the Solanaceae family, with an emphasis on the genus *Capsicum.* These peptides display significant biological activity against pathogens, demonstrating their therapeutic potential. Within the *Capsicum* genus, AMPs, such as LTPs, defensins, and protease inhibitors, which exhibit antifungal and antibacterial activities, have been characterized. The extensive range of AMPs present in the Solanaceae family provides insight into the development of medical and agricultural products, and ongoing research continues to unravel their potential applications.

## 4. Toxicity and Potential Applications of Plant AMPs

Although AMPs are interesting alternatives for drug development, few studies with this objective have advanced to the preclinical phase of animal testing. The physical and chemical instability of peptides, short half-life, proteolytic degradation and pharmacokinetic and pharmacodynamic characteristics of AMPs are the main obstacles to their clinical application [194,195]. More than 3000 AMPs have been identified, but few have advanced to the clinical testing phase. A few enzymatically synthesized antimicrobial peptides, such as gramicidins and polymyxins, are approved by the U.S. Food and Drug Administration (FDA), and most of them are used for topical use in bacterial and fungal skin infections [196,197]. Currently, a defensin derivative named Pezadeftide is the only example of plant AMP that is in the preclinical trial. This compound is being developed by Hexima Limited company for the treatment of nail mycosis with excellent clinical efficiency. Other AMPs are in the clinical testing phase but are not of plant origin. Brilacidin is a synthetic defensin derivative that is being developed for the treatment of oral mucositis in patients with head and neck cancer, and Plectasin is a defensin variant that is being developed for the treatment of infections caused by Gram-positive bacteria [198].

Toxicity to mammalian cells is the main bottleneck for the approval of bioactive peptides [199]. Finding a balance between AMP bioactivity and patient safety is the main challenge for drug development. However, AMPs of natural origin can serve as a framework for the development of drugs with reduced toxicity, maintaining or increasing their bioactivities. To achieve this goal, synthetic peptides can be designed to have greater selective toxicity by adjusting their amino acid composition and peptide chain structure, or AMPs can be conjugated with other molecules, such as biopolymers, magnetic nanoparticles, liposomes, and other compounds, for the creation of drug delivery systems. Furthermore, the emergence of pathogens and pests resistant to these peptides is rare, due to their target specificity (cell membranes) and their rapid action. From the amino acid sequence and structural information of naturally occurring AMPs, a new generation of short synthetic AMPs can be obtained using bioinformatics tools for therapeutic purposes, with better selectivity and biocidal activity. Various new strategies such as cyclization, hydrophobicity modifications, and the incorporation of non-natural amino acids have enormous potential to improve the stability of AMPs while maintaining or improving their selective toxicity [200].

## 5. Conclusions

Notably, in the last two decades, the number of studies involving plant AMPs has increased. In this scenario, AMPs isolated from plants of the genus *Capsicum* of the Solanaceae family exhibit mainly antifungal activities and enzymatic inhibition activities, and it is necessary to better investigate their potential for other biological activities. This review elucidates the potential for the production of bioactive compounds by plant AMPs, highlighting new knowledge about their bioactive properties. In addition, this study provides new perspectives for the study of plant AMP families and their potential in the treatment of infections caused by pathogens of medical and agricultural interest, because microorganisms are less likely to develop resistance to such compounds. Thus, the study of plant AMPs contributes to increasing food productivity and the development of new therapies to improve the population’s quality of life.

## Figures and Tables

**Table 1:** Compilation of representative peptides from plant thionins, hevein-like peptides, defensins, LTPs, and cyclotides.

Thionins	Plant	Biological Activity	Main Target	Reference
Asthi1	*Oryza sativa* L.	Antibacterial	*Burkholderia plantarii*	[35]
Thionin Thi2.1	*Arabidopsis thaliana* L.	Antibacterial, antifungal, and cytotoxic	*Staphylococcus aureus*, *Escherichia coli*, *Candida albicans*; various mammalian cell lines	[36]
Thionin Thi2.1 (transgenic)	*Lycopersicon esculentum* Mill.	Antimicrobial	*Ralstonia solanacearum* and *Fusarium oxysporum* f. sp. *lycopersici*	[37]
Barley thionin	*Ipomoea batatas* (L.) Lam.	Antifungal	*Ceratocystis fimbriata*	[38]
NsW1 and NsW2	*Nigella sativa* L.	Antifungal and Cytotoxic	*Aspergillus ochraceus* and *A. fumigatus*; RD, Jukart cell lines	[39]
Cp-thionin	*Vigna unguiculata* L.	Trypsin inhibitor	Trypsin	[40]
Thionin	*Nicotiana tabacum*	Antifungal and antibacterial	*Botrytis cinerea*, *Ralstonia* *solanacearum*, *Helicoverpa armigera*	[41]
Thionin	*Allium cepa*	Antifungal	*Aspergillus niger*	[42]
Barley thionin	*Nicotiana benthamiana* Domin.	Antimicrobial and insecticidal	*Myzus persicae*	[43]
Pp-TH	*Pyrularia pubera* M.	Antifungal and antibacterial activities anticoagulant	*R. meliloti*, *X. campestris* pv. *translucens*, and *X. campestris* pv. *campestris*, *C. michiganensis*; *Pseudonebularia cucumerina*, *F. oxysporum*, and *B. cinerea*	[44,45]
Ligatoxin B	*Phoradendron liga* (Gill.) Eichl	Cytotoxic	Human lymphoma cell line U-937-GTB and the primary multidrug-resistant renal adenocarcinoma cell line ACHN	[46]
Phoratoxins C-F	*Phoradendron tomentosum* (DC.) Engelm. ex A.Gray	Antitumoral	Solid tumor and hematological tumor	[47]
β-purothionin	*Triticum kiharae*	Antimutagenic	Human RD cells; DNA protection from cadmium chloride	[48]
Thionin Thi2.1	*Arabidopsis thaliana* L.	Antibacterial, antifungal, and cytotoxic	*Staphylococcus aureus*, *Escherichia coli*, *Candida albicans*; various mammalian cell lines	[36]
Thionin-like AMPs	*Arabidopsis thaliana* L.	Nematocidal	*Heterodera schachtii*	[36]
Mthionin	*Arabidopsis thaliana* L.	Antifungal	*Fusarium graminearum*	[49]
PsoTHI1.7	*Papaver somniferum* L.	Antifungal	*Fusarium oxysporum* f. sp. *matthiolae* and *Botrytis cinerea*	[50]
Tu-MP 1 (a) and Tu-MP 2 (b)	*Tulipa gesneriana* L.	Antifungal and antibacterial	*P. carotovora*, *A. radiobacter*, *A. rhizogenes*, *C. michiganensis*, and *C. flaccumfaciens*; *F. oxysporum* and *G. candidum*	[51]
Thionin	*Oryza sativa* L.	Nematocidal	*Meloidogyne graminicola*, *Pythium graminicola*	[52]
**Heveins**	**Plant**	**Biological** **Activity**	**Main Target**	**Reference**
Ee-CBP	*Euonymus europaeus* L.	Antifungal	*Botrytis cinerea*	[53]
Hevein-like AMPs	*Eucommia ulmoides* Oliv.	Antifungal	*Phytophthora infestans*, *Aculops lycopersicu*,	[54]
			*Verticillium dahliae*, *Gibberella zeae*,	
			*Alternaria nicotianae*, *Fusarium moniliforme*,	
			*Fusarium oxysporum*, *Corythucha gossypii*	
Hb-AMP1	*Hevea brasiliensis* Muell. Arg.	Antifungal and antibacterial	*Candida albicans*, *Candida tropicalis*, *Candida krusei*, *Porphyromonas gingivalis*, *Prevotella intermedia*, *Tannerella forsythia*, *Aggregatibacter* *actinomycetemcomitans*	[55]
Hevein-like AMPs	*Broussonetia papyrifera* (L.) L’Hér. ex Vent., *Morus papyrifera* L.	Antifungal	*Trichoderma viride*	[56]
Sm-AMP-X	*Stellaria media* L.	Antifungal and antibacterial	*Fusarium solani*, *Alternaria* *alternata*, *Bipolaris sorokiniana*, *Botrytis cinerea*, *Aspergillus niger*, *Escherichia coli*, *P. carotovora*, *Clavibacter michiganensis* pv. *michiganensis*, *Agrobacterium rhizogenes*, *Bacillus subtilis*, *Pseudomonas syringae* pv. *tomato*	[57]
Pn-AMP1 and Pn-AMP2	*Pharbitis nil* L.	Antifungal	*Candida albicans*, *Candida tropicalis*, *Candida krusei*, *Phytophthora capsici*	[58]
Hevein-like genes	*Hevea brasiliensis* Muell. Arg.	Antifungal	*Magnaporthe grisea*	[59]
Wj-AMP	*Wasabia japonica* L.	Antifungal and antibacterial	*Botrytis cinerea*, *Pseudomonas cichorii*, *Pseudomonas glumae*, *Pseudomonas plantaii*, *Agrobacterium tumefaciens*, *Escherichia coli*	[60]
Sm-AMP	*Stellaria media* L.	Antifungal	*Bipolaris sorokiniana*, *Thielaviopsis basicola*	[61]
Pro-SmAMP1	*Stellaria media* L.	Antifungal	*Alternaria* spp.	[62]
Hevein-like genes	*Linum usitatissimum* L.	Antifungal	*Fusarium oxysporum*	[63]
Hevein-like peptides	*Moringa oleifera*	Coagulation/ flocculation activities	Highly efficient potential of peptides	[64]
**Defensins**	**Plant**	**Biological** **Activity**	**Main Target**	**Reference**
PaDef1	*Persea americana* *var. drymifolia* Mill.	Anticancer	Jurkat lineage of acute lymphocytic leukemia cells	[65]
DmDef1	*Dahlia merckii* Lehm.	Antifungal	*Saccharomyces cerevisiae*	[66]
DmDef2, DmDef3	*Dahlia merckii* Lehm.	Antifungal	*Botrytis cinerea*, *Verticillium albo-atrum*	[67]
MtDef1, MtDef2	*Medicago truncatula* Gaertn.	Antibacterial and antifungal	*Escherichia coli*, *Pseudomonas syringae* pv. *syringae*, *Sinorhizobium meliloti*, *Xanthomonas alfalfae* subsp. *alfalfae*, *Phytophthora medicaginis*, *Fusarium solani*	[68]
NaDef1, NaDef2	*Nicotiana alata* Link and Otto	Antifungal and antibacterial	*Fusarium oxysporum*, *Botrytis cinerea*	[69]
FeDef1, FeDef2	*Fagopyrum esculentum* Moench.	Antibacterial and antifungal	*Clavibacter michiganensis*, *Curtobacterium flaccumfaciens*, *P. carotovora*, *Agrobacterium* spp., *Fusarium oxysporum*, *Geotrichum candidum*	[70]
MsDef1	*Medicago sativa* L.	Antifungal	*Fusarium graminearum*	[71]
VaDef1, VaDef2	*Vigna angularis* (Willd.) Ohwi and H. Ohashi	Antibacterial and antifungal	*Fusarium oxysporum*, *Fusarium* sp. *pisi*, *Trichophyton rubrum*, *Staphylococcus epidermidis*, *Bacillus cereus*, *Xanthomonas campestris* pv. *vesicatoria*	[72]
PlDef1	*Phaseolus limensis* L.	Antifungal and antibacterial	*Botrytis cinerea*, *Fusarium oxysporum*, *Mycosphaerella arachidicola*	[73]
RsAFP2	*Raphanus sativus*	Antifungal	*Candida albicans*, *Pichia pastoris*	[74]
BcDef1	*Brassica campestris* L. ssp. pekinensis	Antifungal	*Alternaria solani*, *Neurospora crassa*, *Phytophthora parasitica*, *Fusarium oxysporum*	[75]
OsDef1	*Oryza sativa* cv. Sasanishiki	Antifungal	*Magnaporthe grisea*	[76]
TaDef1	*Triticum aestivum* L.	Antibacterial	*Pseudomonas cichorii*	[77]
VrDef1	*Vigna radiata* (L.) Wilczek	Insecticidal and antifungal	*Callosobruchus chinensis* larvae, *Fusarium oxysporum*, *Pyricularia oryzae*, *Rhizoctonia solani*, *Trichophyton* *rubrum*	[78]
VuDef1	*Vigna unguiculata*	Enzymatic inhibitor	*Acanthoscelides obtectus*, *Zabrotes subfasciatus*	[79]
PvDef1	*Phaseolus vulgaris* cv. Pérola	Antifungal and antibacterial	*Candida albicans*, *Candida* *parapsilosis*, *Candida tropicalis*, *Candida guilliermondii*, *Kluyveromyces marxianus*, *Saccharomyces cerevisiae*, *Fusarium oxysporum*, *Fusarium* *solani*, *Fusarium lateritium*, *Rhizoctonia solani*	[80]
PeDef1	*Pachyrhizus erosus* (L.) Urb.	Antifungal	*Fusarium oxysporum* f. sp. *vasinfectum*, *Verticillium dahliae*, *Aspergillus flavus*, *Penicillium* spp., *Colletotrichum* *gloeosporioides*, *Botrytis cinerea*, *Bipolaris maydis*, *Aspergillus niger*, *Fusarium oxysporum* f. sp. *lycopersici*, *Rhizopus stolonifer*	[81]
LmDef1	*Lepidium meyenii*	Antimicrobial	*Phytophthora infestans*	[82]
PsDef1	*Pisum sativum*	Antifungal	*Aspergillus niger*	[83]
TfDef1	*Trigonella foenum-graecum*	Antifungal	*Rhizoctonia solani*, *Phaeoisariopsis personata*	[84]
MsDef2	*Medicago sativa* L.	Antifungal	*Fusarium oxysporum* f. sp. *lycopersici*	[85]
NaDef3	*Nicotiana alata*	Antifungal	*Fusarium oxysporum*, *Verticillium dahliae*	[86]
RsAFP2	*Raphanus sativus*	Antifungal	Specific amino acid region affecting activity (RsAFP2)	[87]
MsDef1, MtDef4	*Medicago sativa*, *Medicago truncatula*	Antifungal	Specific amino acid region affecting activity (MsDef1, MtDef4)	[88]
**LTPs**	**Plant**	**Biological** **Activity**	**Main Target**	**Reference**
LTP	*Coffea canephora* L.	Antifungal and antibacterial	*Colletotrichum lindemuthianum*, *Colletotrichum gloeosporioides*, *Fusarium solani*, *Fusarium lateritium*, *Colletotrichum* sp., *Xanthomonas euvesicatoria*	[80]
LTP-like AMPs	*Capsicum annuum* L. cv. Bugang	Antibacterial and antiviral	*Tobacco mosaic virus* (TMV), *Xanthomonas campestris* pv. *vesicatoria*	[82,83]
LTP	*Oryza sativa*	Antimicrobial	*Pyricularia oryzae*, *Xanthomonas oryzae*	[84]
nsLTP AMPs	*Phaseolus mungo*	Antifungal and antibacterial	*Fusarium solani*, *Fusarium* *oxysporum*, *Pythium* *aphanidermatum*, *Sclerotium rolfsii*, Gram-positive bacteria *Staphylococcus aureus*	[85]
LTP	Potato plants	Antimicrobial	*Phytophthora infestans*	[86]
nsLTP-like AMPs	*Leonurus japonicus* Houtt.	Antifungal and antibacterial	*Fusarium oxysporum*, *Alternaria* *brassicae*, *Bipolaris maydis*, *Rhizoctonia* *cerealis*, *Saccharomyces cerevisiae*, *Bacillus* *subtilis*	[87]
LTP-like AMPs	*Brassica campestris* L.	Antifungal	*Fusarium oxysporum*, *Mycosphaerella arachidicola*	[88]
LTP	*Helianthus annuus*	Antifungal	*Fusarium solani* f. sp. *eumartii*, *Alternaria alternata*	[89]
LTP-like AMPs	*Narcissus tazetta* var. *chinensis* L.	Antiviral	Respiratory syncytial virus (RSV), Influenza A (H1N1) virus	[90]
LTP	*Nigella sativa* L.	Antifungal	*Pythium debaryanum*, oomycetes *Phytophthora infestans*	[91]
LTP-like AMPs	*Coffea canephora*	Antifungal	*Candida albicans*, *Candida tropicalis*, inhibition of α-amylase in mammals	[92]
nsLTP-like AMPs	Transgenic wheat	Antifungal	*Fusarium graminearum*	[93]
LTP-like AMPs	Wheat	Antifungal	*Alternaria* sp., *Rhizoctonia solani*, *Curvularia lunata*, *Bipolaris oryzae*, *Cylindrocladium scoparium*, *Botrytis cinerea*, *Sarocladium oryzae*	[94]
LTP-like AMPs	*Anethum graveolens* L.	Antifungal	Weak antifungal activity, inhibition of spore germination, delay in hyphae elongation of *Aspergillus niger*	[95]
LTP	*Allium cepa* L.	Antifungal	*Blumeria graminis* f. sp. *tritici*, *Neovossia indica*	[96]
LTP	*Triticum aestivum*	Antifungal	*Puccinia triticina* Erikss.	[97]
**Cyclotides**	**Plant**	**Biological** **Activity**	**Main Target**	**Reference**
kalata B1, kalata B2, cycloviolacin O2	*Viola arvensis* Murr., *Viola odorata* L.	Antitumor and cytotoxic effects	Human tumor cell lines	[98,99]
hylin C, hylin D	*Hybanthus enneaspermus* (L.) F. Muell.	Anticancer, aphrodisiac properties	Erectile function in male rats, membrane blistering and cell necrosis	[100,101]
rin A, rin B	*Rinorea spp.*	Cytotoxicity	Pathogenic bacteria and possibly cancer cells	[102]
pom A, pom B	*Pombalia calceolaria*	Anticancer	Human tumor cell lines	[103]
varv A, varv F	*Viola tricolor*	Inhibitory activity against HIV	Human immunodeficiency virus (HIV)	[104]
vhl-1	*Viola hederacea*	Inhibitory activity against HIV	Human immunodeficiency virus (HIV)	[105]
viy A	*Viola yedoensis* Makino	Anti-HIV	Human immunodeficiency virus (HIV)	[106]
Cyclotides	*Viola odorata*	Anti-inflammatory, reduces demyelination	Inflammation, neurological deficits in experimental autoimmune encephalomyelitis model	[107]
Cyclotides	*Oldenlandia affinis*	Anti-inflammatory, antiplasmodial	*Plasmodium berghei*, acute and chronic inflammation in rodent models	[108]
Cyclotides	*Allexis* spp.	Protease inhibitor	Inhibition of human prolyl oligopeptidase activity	[109]
SNC1 and SNC2	*Sambucus nigra*	Antibacterial	*Aeromonas salmonicida*, *Vibrio ordalii*, *Vibrio anguillaru*, *Flavobacterium psychrophilum*, *E. coli*	[110]
cycloviolacin O2	*Viola tricolor*	Antibacterial	*Pseudomonas aeruginosa* and *Staphylococcus aureus, Escherichia coli and Bacillus subtilis.*	[111]
Cyclotides	*Viola odorata*	Antibacterial	*Staphylococcus aureus*	[112]
Cyclotides	*Geophila repens*	Antibacterial and cytotoxic activity	*Escherichia coli*, U-937 human lymphoma cell line	[113]
Kalata B1, Kalata B2, OlaC1, OlaC2	*Oldenlandia affinis*	Insecticidal	Moth larvae *Helicoverpa armigera*	[114,115]
Cyclotides	*O. affinis*, *Clitoria ternatea*, *Viola odorata*, *Hybanthus enneaspermus*	Nematocidal effects	*Caenorhabditis elegans* juvenile	[116]
